# Production of CXC and CC Chemokines by Human Antigen-Presenting Cells in Response to Lassa Virus or Closely Related Immunogenic Viruses, and in Cynomolgus Monkeys with Lassa Fever

**DOI:** 10.1371/journal.pntd.0002637

**Published:** 2014-01-09

**Authors:** Delphine Pannetier, Stéphanie Reynard, Marion Russier, Xavier Carnec, Sylvain Baize

**Affiliations:** 1 Unité de Biologie des Infections Virales Emergentes, Institut Pasteur, Lyon, France; 2 Laboratoire P4 Inserm-Jean Mérieux, US003, Lyon, France; 3 Centre International de Recherche en Infectiologie (CIRI), Université de Lyon, INSERM U1111, Ecole Normale Supérieure de Lyon, Université Lyon 1, CNRS UMR5308, Lyon, France; 4 Unité de Génétique Moléculaire des Bunyavirus, Institut Pasteur, Paris, France; University of Texas Medical Branch, United States of America

## Abstract

The pathogenesis of Lassa fever (LF), a hemorrhagic fever endemic to West Africa, remains unclear. We previously compared Lassa virus (LASV) with its genetically close, but nonpathogenic homolog Mopeia virus (MOPV) and demonstrated that the strong activation of antigen-presenting cells (APC), including type I IFN production, observed in response to MOPV probably plays a crucial role in controlling infection. We show here that human macrophages (MP) produce large amounts of CC and CXC chemokines in response to MOPV infection, whereas dendritic cells (DC) release only moderate amounts of CXC chemokines. However, in the presence of autologous T cells, DCs produced CC and CXC chemokines. Chemokines were produced in response to type I IFN synthesis, as the levels of both mediators were strongly correlated and the neutralization of type I IFN resulted in an inhibition of chemokine production. By contrast, LASV induced only low levels of CXCL-10 and CXCL-11 production. These differences in chemokine production may profoundly affect the generation of virus-specific T-cell responses and may therefore contribute to the difference of pathogenicity between these two viruses. In addition, a recombinant LASV (rLASV) harboring the NP-D389A/G392A mutations, which abolish the inhibition of type I IFN response by nucleoprotein (NP), induced the massive synthesis of CC and CXC chemokines in both DC and MP, confirming the crucial role of arenavirus NP in immunosuppression and pathogenicity. Finally, we confirmed, using PBMC samples and lymph nodes obtained from LASV-infected cynomolgus monkeys, that LF was associated with high levels of CXC chemokine mRNA synthesis, suggesting that the very early synthesis of these mediators may be correlated with a favourable outcome.

## Author Summary

Lassa virus (LASV) causes a viral hemorrhagic fever that affects about 300,000 people and leads to 5,000 deaths annually. Lassa fever (LF) is a public health problem in West Africa, where it is endemic, because of the number of cases, deaths and disabling effects. There is no vaccine against LASV and the only treatment, ribavirin, is not useful in the field. Little is known about the pathogenesis and immune responses associated with LF. Chemokines are involved in the induction of immunity and attraction of immune cells to inflamed sites. We compared the ability of antigen-presenting cells to produce chemokines in response to infection with LASV, the closely related but nonpathogenic Mopeia virus (MOPV) and a LASV unable to inhibit the type I IFN response due to mutations in its nucleoprotein gene. We found that MOPV and the mutant LASV, but not wild-type LASV, strongly induced CC and CXC chemokine production by dendritic cells and macrophages, in a type I IFN-dependent manner. We confirmed in cynomolgus monkeys that these mediators probably play a role during LF. These results highlight the role of innate immunity in LF control and provide insight into the mechanisms leading to survival or death after infection.

## Introduction

Lassa virus (LASV) is the causal agent of Lassa fever (LF), a hemorrhagic fever endemic to West Africa [1]. The virus is transmitted to humans through contact with infected *Mastomys* sp., rodents living close to housing and constituting a natural reservoir of LASV. Human-to-human transmission then occurs through mucosal/cutaneous contact. LF affects about 300,000 people each year, resulting in 5,000–6,000 deaths. There is no approved vaccine against the disease, and the only treatment available, ribavirin, is neither fully effective nor useful in the field, due to its limited availability and the need to initiate treatment soon after infection [2]. LF is therefore a major public health concern in the countries in which it is endemic, and this problem is exacerbated by the tendency of the zone of endemicity to expand [3].

LASV is an Old World arenavirus from the *Arenaviridae* family. It is an enveloped bisegmented RNA virus. Its small segment (S) encodes the nucleoprotein (NP) and the glycoprotein precursor (GPC) and is cleaved by the subtilase SKI-1/S1P to generate GP_1_ and GP_2_, mediating viral entry by binding to α-dystroglycan [4], [5]. The large segment (L) encodes the RNA-dependent RNA polymerase and the Z protein, a small zinc-binding protein important for replication, transcription and viral budding [6], [7], [8], [9]. The pathogenesis of LF is poorly understood. Antigen-presenting cells (APC), dendritic cells (DC) and macrophages (MP) are the principal initial targets of LASV [10], [11], [12]. The first few cycles of viral replication occur in these cells and the tropism of LASV then widens, such that viral replication also occurs in hepatocytes, endothelial cells, fibroblasts and some epithelial cells [13], [14]. However, changes to the liver, endothelium and other organs are not severe enough to account for death, which occurs in a context of hypoxic, hypotensive and hypovolemic shock. Little is known about the immune responses associated with survival or death after LF. The production of specific antibodies (Ab) is not correlated with survival, as such Ab are detected in all patients, regardless of outcome [15]. Moreover, LASV does not induce the production of neutralizing Ab [16], [17]. Instead, protection seems to depend on the induction of specific T-cell responses [17], [18], [19].

Mopeia virus (MOPV) is an Old World arenavirus closely related to LASV. Indeed, MOPV is 60 to 80% identical to LASV in terms of its nucleotide and amino-acid sequences and has also been isolated from *Mastomys natalensis* [20], [21], [22]. However, MOPV is nonpathogenic in non human primates (NHP), and probably also in humans, and it can even immunize monkeys against LASV [23]. We investigated the immune responses associated with LF, by comparing the responses induced by these two viruses in human *in vitro* models. The infection of DC leads to the release of large amounts of LASV and MOPV, without significant cell activation or cytokine production [10], [24], [25]. MP were not activated by LASV infection, but strong activation and significant amounts of type I IFN production were observed in response to MOPV infection. We showed that viral tropism for APC probably played a crucial role in pathogenesis. Indeed, MOPV-, but not LASV-, infected DC induce robust primary human T-cell responses *in vitro* [26].

The lack of induction of type I IFN production by LASV is due to the presence in the C-terminal part of the NP of a dsRNA-specific 3′ to 5′ exonuclease related to the enzymes of the DEDDh family [27], [28]. By digesting viral dsRNA, LASV escapes recognition by the RIG-I and MDA-5 helicases, which have been implicated in the sensing of arenavirus RNA and the induction of type I IFN production [29]. The amino-acid residues involved in this activity have been described and their mutation abolishes the anti-IFN properties of NP [30]. The differences in pathogenicity and type I IFN production between LASV and MOPV cannot be due to exonuclease activity alone, as the DEDDh motif is also present in the MOPV NP. The MOPV NP therefore probably also inhibits type I IFN production, albeit less efficiently than the LASV NP. We therefore used reverse genetics to generate a rLASV containing mutations affecting the DEDDh motif. This virus induced a much stronger type I IFN response than MOPV [31]. In addition, it has recently been shown that the arenavirus NP inhibits IRF3 phosphorylation and subsequent type I IFN synthesis by sequestering the IκB kinase-related kinase IKKε in an inactive form [32]. Finally, arenavirus NP has also been reported to prevent the nuclear translocation of NFκB, consistent with the lack of proinflammatory cytokine release observed in response to LASV infection [33].

Inflammatory chemokines are crucial mediators in the development of innate and adaptive immune responses and viral control, but they may also participate in pathogenesis [34], [35], [36], [37], [38]. The role of chemokines in LF is poorly understood. The release of CXCL10 (IP-10) and IL-8 into plasma has been correlated with survival in LASV-infected patients [39]. Consistent with this finding, LASV-infected human monocytes/MP, unlike their MOPV-infected counterparts, fail to produce IL-8 [40]. In addition, no increase in plasma concentrations of CCL5 (RANTES), CXCL9 (MIG), CXCL-10, and IL-8 has been detected in moribund LASV-infected cynomolgus macaques; CCL2 (MCP-1) was the only chemokine circulating in significant quantities in these animals [11]. In another study in cynomolgus macaques, increases in the synthesis of CXCL-10 and CXCL11 (I-TAC) mRNA were reported in PBMC and lymph nodes, regardless of the outcome of infection [17]. Finally, rhesus macaques intravenously infected with LCMV as a surrogate for LF display high plasma concentrations of IL-8 and CXCL10, together with strong synthesis of CXCL10 mRNA in PBMC [41]. We compared the production of CC and CXC chemokines by human DC and MP in response to infection with LASV and MOPV, but also in response to infection with LASV-NP D389A/G392A, a rLASV harboring mutations affecting the NP exonuclease, preventing it from inhibiting type I IFN production. We also confirmed that cynomolgus monkeys produced large amounts of CXC chemokines *in vivo* during LF.

## Materials and Methods

### Viruses and cell lines

The AV strain of LASV, isolated from the serum of a patient [42], and MOPV, strain AN 23166, isolated from *Mastomys natalensis* [22], were subjected to four passages on Vero E6 cells at 37°C, under an atmosphere containing 5% CO_2_, in Dulbecco's modified Eagle medium supplemented with 50 IU/ml penicillin-streptomycin, 1% non essential amino acids (all from Invitrogen, Cergy-Pontoise, France) and AB+ human serum (*Etablissement Français du Sang* [EFS], Lyon, France). Cell-free supernatants were harvested after three days for LASV infection and after four days for MOPV infection, and were used as infectious virus stocks, with a viral titer of 2.5×10^7^ FFU/ml. The rLASV NP-D389A-G392A and wild-type rLASV were generated by reverse genetics techniques, as previously described [31], and were passaged twice on Vero E6 cells to obtain the viral stocks (1.2×10^7^ FFU/ml). BSL-4 facilities (Laboratoire P4-INSERM Jean Mérieux, Lyon) were used for all experiments with LASV, whereas MOPV was manipulated in BSL-2 facilities. Vero E6 cells and virus stocks were not contaminated with mycoplasma.

### Preparation of DC and MP

Monocytes and lymphocytes were isolated from the blood of healthy human donors (EFS), as previously described [10], [26]. Briefly, peripheral blood mononuclear cells (PBMC) were isolated by density gradient centrifugation on Ficoll-Paque (GE-Healthcare BioSciences AB). Autologous plasma (AP) was harvested, heated for 30 min at 56°C and centrifuged for T cell experiments. Monocytes were then separated from peripheral blood lymphocytes (PBL) by centrifugation on 50% Percoll (GE-Healthcare) in PBS and purified by immunomagnetic depletion. PBL were frozen in RPMI 1640-Glutamax I with 50 IU/ml penicillin-streptomycin, 1% non essential amino acids, 10 mM HEPES (C-RPMI) supplemented with 20% AP and 10% dimethylsulfoxide (DMSO) (Sigma-Aldrich, Saint-Quentin Fallavier, France) and 20% AP and stored in liquid nitrogen. Cells were cultured in C-RPMI supplemented with either 10% FCS for APC cultured alone or 10% AP for cultures containing autologous T cells in order to prevent non specific stimulations (full-RPMI). iDC and MP were differentiated from monocytes in full-RPMI supplemented with 2000 IU/ml recombinant human (rh) granulocyte-macrophage colony-stimulating factor plus 1000 IU/ml rh interleukin (IL)-4 or with 10 ng/ml rh macrophage colony-stimulating factor, respectively (all from PeproTech, Rocky Hill, NJ, USA). Half the cytokine content and 40% of the culture medium were replaced every 48 h and cells were harvested six days later. In experiments using T cells, two vials of DC were frozen in AP containing 10% DMSO for subsequent re-stimulation.

### Infection of DC and MP with LASV

DC and MP cell pellets were incubated with virus-free Vero E6 cell supernatant (mock infection) or infectious LASV or MOPV at a multiplicity of infection of 2, for 1 h at 37°C, with regular shaking. DC and MP were then washed and cultured (10^6^ cells/ml) in full-RPMI. In some experiments, the type I IFN receptor was neutralized by adding antagonistic mAbs directed against CD118, the beta chain of this receptor (5 µg/ml, PBL Biomedical Laboratories, Piscataway, NJ).

### Coculture of DC and T cells *in vitro*

We previously described an *in vitro* model of DC and T-cell co-culture, with three rounds of stimulation [26]. The first round of stimulation involved the infection of DC with infectious LASV or MOPV. DC were then cocultured with autologous T cells at a ratio of 1 DC to 10 T lymphocytes, at a density of 2×10^5^ and 2×10^6^ cells/ml, respectively. PBL were thawed and depleted of B and NK cells as previously described [26]. The second and third rounds of stimulation were carried out 9 and 19 days after the first round of stimulation by culturing thawed iDC stimulated by inactivated viruses or culture medium (mock) and T cells harvested from the previous stimulation. We replaced 30% of the culture medium with fresh medium every two or three days. On day 2 of each round of stimulation, 10 IU/ml (for the first stimulation) or 5 IU/ml (second and third stimulations) of rhIL-2 and rhIL-7 (both from Peprotech) were added to the culture medium. In some experiments, the type I IFN receptor was neutralized by adding 5 µg/ml antagonistic mAbs directed against CD118 (PBL Biomedical Laboratories) every two days during the first round of stimulation. Control cells were treated similarly, with an irrelevant mouse IgG2a (R&D Systems, Lille, France).

### Detection of mRNA by RT-PCR

Cellular RNAs were isolated from human cells and cynomolgus monkey PBMC or lymph node cells and used for the synthesis of first-strand cDNA as previously described [10], [17]. In human samples, mRNAs encoding cytokines were quantified by real-time RT-PCR (RT-qPCR) with commercially available primers and probes and TaqMan Universal Master Mix on an ABI PRISM 7000 real-time thermocycler and cDNA were amplified in duplex with β-actin or GAPDH (all from Applied Biosystems). Other TaqMan assays were carried out with the following primers and probes: IFNβ: 5′-TCTCCACGACAGCTCTTTCCA-3′ and 5′-ACACTGACAATTGCTGCTTCTTTG-3′, probe: 5′-AACTTGCTTGGATTCCT-3′ ; IFNα1: 5′-GTGGTGCTCAGCTGCAAGTC-3′ and 5′-TGTGGGTCTCAGGGAGATCAC-3′, probe: 5′-AGCTGCTCTCTGGGC-3′ ; IFNα2: 5′-CAGTCTAGCAGCATCTGCAACAT-3′ and 5′-GGAGGGCCACCAGTAAAGC-3′, probe: 5′-ACAATGGCCTTGACCTT-3′. CCL2, CCL3, CCL5, CXCL9, CXCL10, and CXCL11 mRNAs were quantified in lymph node samples by RT-qPCR in a LightCycler 480 thermocycler (Roche Diagnostic, Meylan, France), using TaqMan Universal Master Mix and commercially available primers and probes (all from Applied Biosystems), as well as the following primers and probe for β-actin: 5′-GCGCGGCTACAGCTTCA-3′ and 5′-CTTAATGTCACGCACGATTTCC-3′, probe: 5′-CACCACGGCCGAGC-3′ . The cDNA obtained from cynomolgus monkey PBMC mRNA was amplified on an ABI Prism 7000 real-time thermocycler (Applied Biosystems) with SYBR green PCR master mix (Applied Biosystems) and the following primers: β-actin: 5′-TGAACCCCAAGGCCAACC-3′ and 5′-GCCAGCCAGGTCCAGACG-3′; CXCL9: 5′-GGAACCCCAGTAATGAGGAAGG-3′ and 5′-GCAGGAAAGGTTTGGAGCAA-3′. CXCL10: 5′-TGAAAAAGAAGGGTGAGAAGAGGT-3′ and 5′-TGATGGCCTTAGATTCTGGATTC-3′; CXCL11: 5′-TACGGTTGTTCAAGGTTTCCC-3′ and 5′-TGGAGGCTTTCTCAATATCTGC-3′. The specificity of the amplicons was checked by determining their melting temperatures.

The levels of cytokine mRNA relative to β-actin or GAPDH mRNA levels for each sample (relative mRNA levels) were calculated as follows:

Δ Cycle threshold (Ct) = Ct gene X - Ct β-actin or GAPDH

The ratio (mRNA of interest/β actin or GAPDH mRNA) = 2 ^−ΔCt^

### ELISA detection of cytokines

Supernatants from cultures of DC and MP were harvested, centrifuged and stored at −80°C. Commercial ELISA kits were used for CCL2, CCL3, CCL4, CCL5, CCL7, CXCL9, CXCL10, and CXCL11 detection, according to the manufacturer's instructions (Bender MedSystems, Vienna, Austria; R&D Systems; BD Biosciences; eBioscience, Paris, France). IFNα was assayed with a human-specific ELISA set (Bender MedSystems), in serial plasma samples obtained from LASV-infected cynomolgus monkeys and control animals [17].

### Infection of cynomolgus monkeys with LASV

Animal experiments were performed in the Jean Mérieux – INSERM BSL4 animal facilities using eight healthy male cynomolgus monkeys (*Macaca fascicularis*) as previously described [17]. All the procedures for animal handling were approved by the “*ethics and animal use committee of the Région Rhône-Alpes*” (record N°007) and performed in accordance with the regulations of the “*European Convention for the Protection of Vertebrate Animals used for Experimental and Other Scientific Purposes*” (ETS n°123, French decree 2001/131). Briefly, six monkeys were infected by low or high doses of LASV as specified and the other two were mock infected and used as negative controls. PBMC were isolated by density gradient centrifugation from blood or inguinal lymph nodes, washed twice and cultured in full-RPMI.

### Statistical analysis

Student's *t*-test and Mann-Whitney were used to compare dataset means. Differences were considered to be significant if the *p* value was less than 0.05. Correlations were assessed by Spearman's rank correlation test. All statistical tests were performed with SigmaPlot software (SyStat Software Inc, San Jose, CA, USA).

## Results

### Antigen-presenting cells produce type I IFN and chemokines in response to MOPV, but not LASV

**Figure 1:**
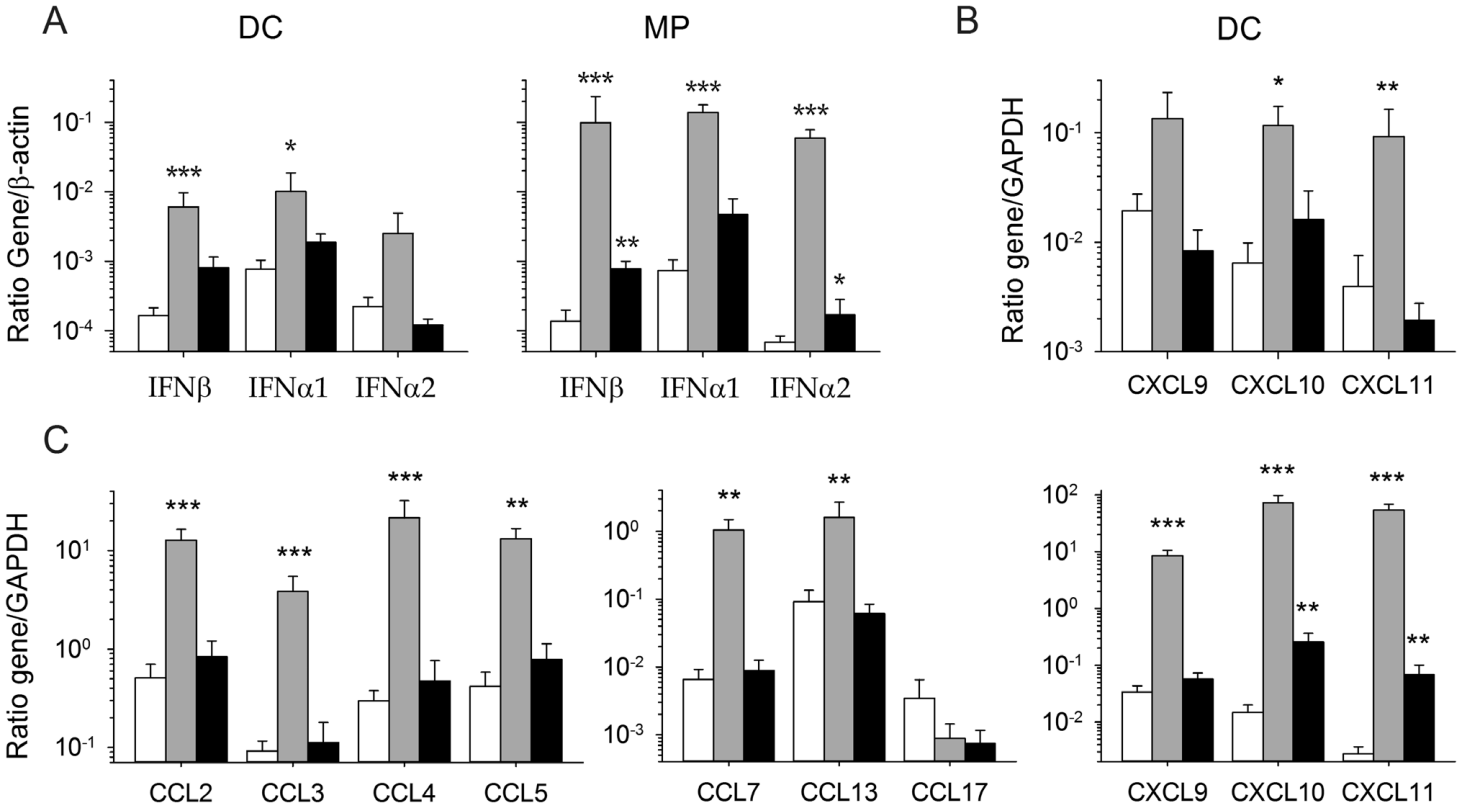
Production of type I IFN and chemokine mRNA in DC and MP infected with LASV and MOPV. We obtained mRNA from DC and MP 24(black bars), MOPV (gray bars), or after mock infection (white bars). (A) The amounts of type I IFN mRNA produced by DC and MP are shown as a gene mRNA/β-actin mRNA ratio. The synthesis of chemokine mRNA was evaluated with the GAPDH gene used as a housekeeping gene, in both DC (B) and MP (C). Results are expressed as the mean ± standard error (SE) of 4 and 5 independent experiments, for LASV- and MOPV-infected cells, respectively. Significant differences between mock- and virus-infected cells are indicated as follows: * (*p*<0.05), ** (*p*<0.01), and *** (*p*<0.001).

Our findings confirm previous reports [24], [25] that MP and, to a lesser extent DC, produce significant amounts of IFNβ, IFNα1 and IFNα2 mRNA in response to MOPV infection. By contrast, LASV-infected DC did not synthesize large amounts of these mRNAs and in LASV-infected MP, only moderate levels are produced (figure 1A). We then quantified the mRNAs encoding CC and CXC chemokines in infected DC and MP. The infection of DC with LASV or MOPV did not lead to synthesis of the CCL2, 3, 4, 5, 7, 13, and 17 mRNAs (data not shown), whereas an increase in the levels of the mRNAs encoding CXCL9, 10, and 11 was observed in MOPV-infected DC, but not in LASV-infected DC (figure 1B). The infection of MP with MOPV induced robust expression of the CCL2, 3, 4, 5, 7, and 13 genes, and of the CXCL9, 10, and 11 genes, in particular (figure 1C). By contrast, the infection of MP with LASV did not result in the transcription of chemokine genes and only CXCL10 and 11 mRNA are significantly produced. Thus, MP and, to a lesser extent, DC, produce type I IFN and chemokines in response to infection with MOPV, but not LASV.

### Macrophages release large amounts of chemokines in response to MOPV

**Figure 2:**
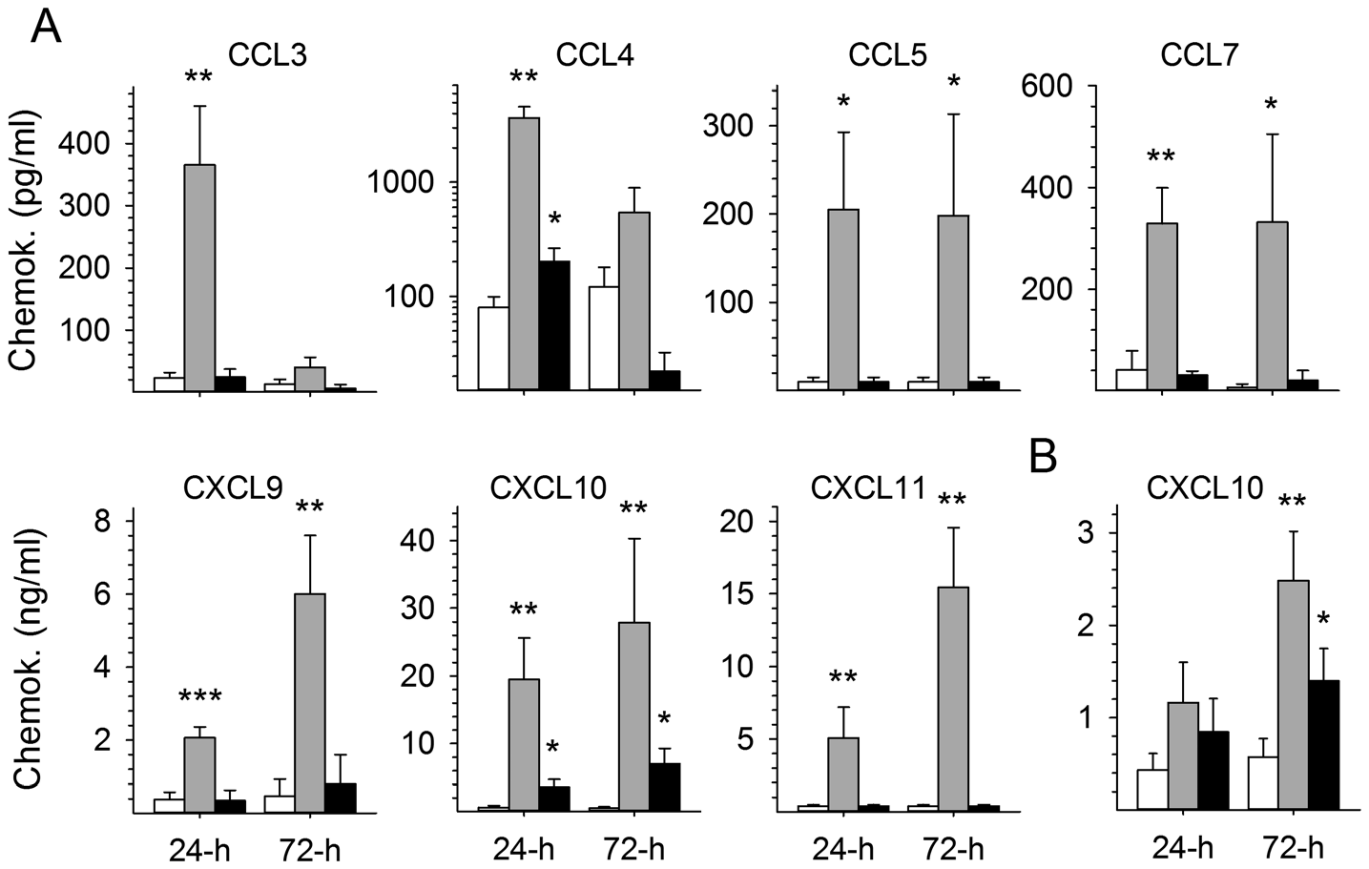
Release of chemokines into the supernatants of DC and MP infected with LASV and MOPV. The levels of CC and CXC chemokines were quantified by ELISA in the supernatants of MP (A) and DC (B) 24 and 72 h after mock (white bars), MOPV (gray bars), and LASV (black bars) infection. Results are expressed in pg/ml and ng/ml for CC and CXC chemokines, respectively. Significant differences are indicated as follows: * (*p*<0.05), ** (*p*<0.01), and *** (*p*<0.001).

The amounts of chemokines released into the culture supernatants were then assessed by ELISA. The results were consistent with mRNA findings, as large amounts of the CC chemokines CCL3, 4, 5, and 7 and, particularly, of the CXC chemokines CXCL9, 10, and 11 were observed 24 and/or 72 h after the infection of MP with MOPV (figure 2A). By contrast, MOPV-infected DC produced only moderate amounts (although significantly higher than those for mock-infected cells) of CXCL10 (data not shown and figure 2B). LASV-infected DC and MP produced only modest amounts of CXCL10, and LASV-infected MP produced only small amounts of CCL4.

### Chemokine production by DC cultured with autologous T cells and relationship to type I IFN production

**Figure 3:**
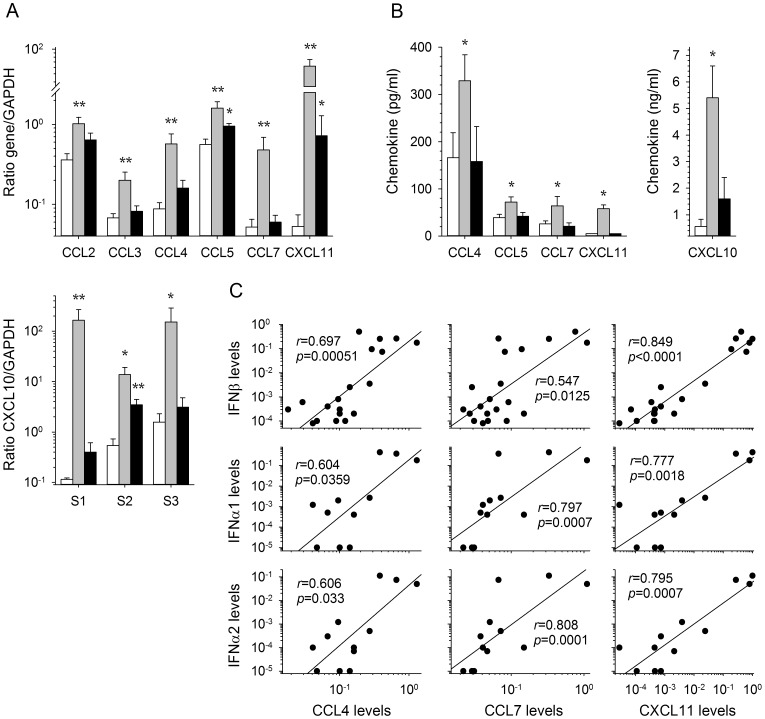
Production of chemokines by MOPV- and LASV-infected DC cocultured with T cells and correlation with type I IFN synthesis. (A) Levels of CC chemokine and CXCL11 mRNA were quantified relative to GAPDH mRNA levels, after 2 days of coculture of mock- (white bars), MOPV- (gray bars), or LASV- (black bars) infected iDC and autologous T cells. Levels of CXCL10 mRNA were evaluated in iDC/T cell coculture 2 days after each stimulation of the T cells with infected or inactivated virus-stimulated iDC. Results are expressed as the mean ± standard error (SE) of 4 independent experiments. Significant differences are indicated as follows: * (*p*<0.05) and ** (*p*<0.01). (B) The levels of CC and CXC chemokines were quantified by ELISA in the supernatants after 2 days of coculture of mock- (white bars), MOPV- (gray bars), or LASV- (black bars) infected iDC and autologous T cells. Results are expressed in pg/ml, except for CXCL10 expressed in ng/ml. Significant differences are indicated as follows: * (*p*<0.05). (C) Correlation between CCL4, CCL7, CXCL10, and CXCL11 mRNA levels and type I IFN synthesis by MOPV- or LASV-infected iDC cultured with naïve T cells 2 days after infection, represented by a linear regression with a correlation coefficient *r* and a probability of correlation *p*.

We recently described an *in vitro* model of the induction of primary human T-cell responses by LASV- and MOPV-infected DC [26]. Using this model, we demonstrated that MOPV-infected DC induced robust CD4^+^ and CD8^+^ T-cell responses involving memory and cytotoxic T cells. By contrast, LASV-infected DC failed to induce significant T-cell responses. We also observed that the presence of T cells in MOPV-infected DC cultures significantly increased the amount of type I IFN produced. We therefore quantified chemokine production in this model. Two days after the first round of stimulation, moderate but significant levels of CCL2, 3, 4, and 5 mRNA were detected in MOPV-infected DC cultured with T cells, but not in LASV-infected DC, other than for CCL5 mRNA synthesis, which was slightly stronger in these cells than in mock-infected cells (figure 3A). CCL7 mRNA levels in MOPV-infected DC were about 10 times higher than those in mock- and LASV-infected DC. Infection with MOPV induced the massive synthesis of CXCL10 and 11 mRNA in DC, whereas only modest levels of CXCL11 mRNA were detected in response to LASV stimulation. By contrast, CXCL9 mRNA levels were not significantly affected by infection with either virus (data not shown). No further significant increase in mRNA synthesis was observed after the second and third rounds of DC stimulation with inactivated viruses (data not shown), other than for CXCL10 mRNA synthesis, which persisted in MOPV-stimulated DC and was significantly induced after the second stimulation with LASV (figure 3A). The levels of CCL4, CCL5, CCL7, CXCL10, and CXCL11 in supernatants were significantly higher in MOPV-infected DC than in mock- or LASV-infected DC cultured with T cells (figure 3B). In contrast, the increase in CCL2 levels in MOPV-infected DC/T cell cocultures was not significant and no difference was observed for CCL3 release in supernatants between mock-, MOPV- and LASV-infected DC/T cell cocultures (data not shown). Finally, the increase in CCL4, CCL7, CXCL10, and CXCL11 expression was strongly correlated with the intensity of IFNβ, α1, and α2 mRNA synthesis in LASV- and MOPV-infected DC (figure 3C).

### Type I IFN is involved in the induction of chemokine production by MOPV-infected DC

**Figure 4:**
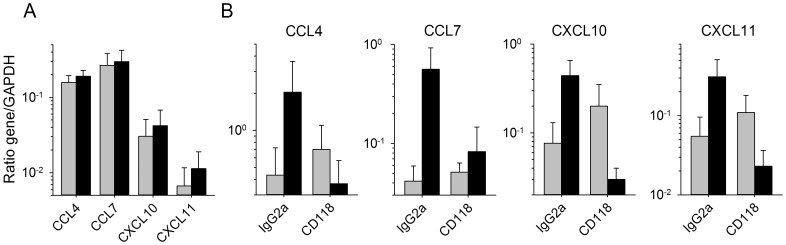
Role of type I IFN in the production of chemokines in MOPV- and LASV-infected iDC/T-cell cocultures. (A) The synthesis of mRNAs for CCL4, CCL7, CXCL10 and CXCL11 was evaluated in mock- (gray bars) or MOPV- (black bars) infected iDC cocultured with naïve T cells for 2 days in the presence of neutralizing Ab against CD118. (B) The levels of these mRNAs then were quantified 2 days after restimulation with mock- (gray bars) or inactivated MOPV- (black bars) pulsed iDC of T cells previously cocultured with mock- or MOPV-infected iDC in the presence of irrelevant IgG2a or CD118-neutralizing Ab. Results are expressed as the mean ± SE of 3 independent experiments.

For confirmation of the involvement of type I IFN in the observed production of chemokines by MOPV-infected DC cultured in the presence of autologous T cells, we neutralized the type I IFN response by adding a neutralizing antibody against CD118, the β chain of the type I IFN receptor, during the first round of T-cell stimulation with MOPV-infected DC and analyzing the synthesis of chemokine mRNA after the first and second stimulations. Neutralization of the type I IFN response abolished the synthesis of the CCL4, CCL7, CXCL10, and CXCL11 mRNAs induced by the first round of stimulation with MOPV-infected DC (figure 4A), and that of the CCL2, CCL3, and CCL5 mRNAs (data not shown). The increase in the synthesis of the CCL4, CCL7, CXCL10 and CXCL11 mRNAs induced by the second round of stimulation with MOPV was not significant, and neutralization of the type I IFN response during the first round of stimulation clearly abolished this overexpression (figure 4B). Similar results were obtained for the CCL2, CCL3, and CCL5 mRNAs (data not shown).

### The recombinant LASV NP-D389A/G392A is a strong inducer of CC and CXC chemokines

**Figure 5:**
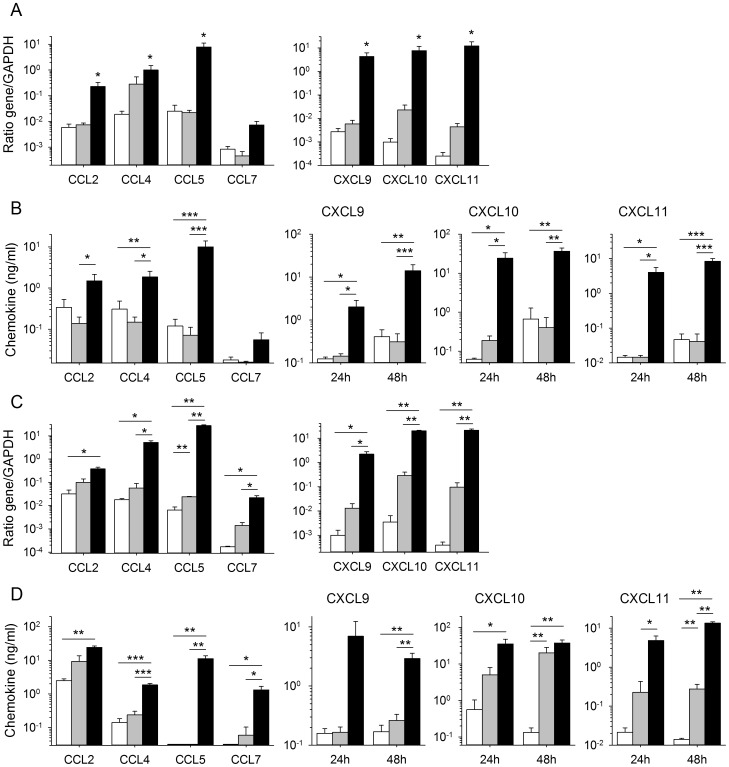
Production of chemokines by APC infected with recombinant LASV. The production of CC and CXC chemokines by DC (A, B) and MP (C, D) was assessed after mock infection (white bars), or infection with recombinant wild-type LASV (gray bars) or rLASV NP-D389A/G392A (rNP LASV) (black bars). (A, C) The synthesis of mRNAs was analyzed by RT-qPCR 24 h after infection. (B, D) The protein levels released in the supernatants were quantified by ELISA 24 h (only for CXCL9, 10 and 11) and 48 h after infection. Results are expressed as the mean ± SE of 4 (A, C) and 3–7 (B, D) independent experiments. Significant differences are indicated as follows: * (*p*<0.05), ** (*p*<0.01) and *** (p<0.001).

We recently described a rLASV harboring mutations affecting the exonuclease site of the NP and abolishing the inhibition of the type I IFN response by LASV [31]. We investigated the involvement of the immunosuppressive properties of the LASV NP in the defective production of chemokines by infected APC, by evaluating chemokine production in NP-D389A/G392A (rNP) LASV-infected APC and comparing the results obtained with those for recombinant wild-type (rWT) LASV. Consistent with the results obtained with LASV (figure 1B and data not shown), no significant increase in the production of CC and CXC chemokine mRNA and proteins was observed in rWT LASV-infected DC (figure 5A and B). By sharp contrast, a robust increase in the amounts of CCL2, CCL4, CCL5, CXCL9, CXCL10, CXCL11 mRNAs was detected in rNP LASV-infected DC and these chemokines were significantly released in large amounts in the culture supernatants. Interestingly, mRNA levels were even higher than those obtained in MOPV-infected DC.

The amounts of CC and CXC chemokines produced were similar in rWT LASV-infected MP (figure 5C and D) and in LASV-infected MP (figure 1C). The infection of MP with rNP LASV led to a robust and significant increase in the synthesis of CCL2, 4, 5, and 7, and of CXCL9, 10, and 11 mRNA and to a strong release of these chemokines in the culture supernatants, as shown by comparison with mock-infected cells. Levels of mRNA and proteins were also significantly higher than those observed in rWT-infected MP, except for CCL2.

### Strong synthesis of CXC chemokines is induced in PBMC and lymph nodes during Lassa fever in cynomolgus monkeys

We investigated whether chemokines were also produced *in vivo* during LF, by evaluating the synthesis of mRNAs encoding CC and CXC chemokines in samples obtained from cynomolgus monkeys infected with LASV. We used mRNA extracted from PBMC obtained from serial blood samples collected during the course of infection and from lymph nodes removed nine days after infection, from three monkeys infected with 10^3^ FFU and three monkeys infected with 10^7^ FFU of LASV. As previously reported, two of the three animals infected with low doses died, 16 and 21 days after infection, whereas all the monkeys infected with high doses survived acute LF [17].

**Figure 6:**
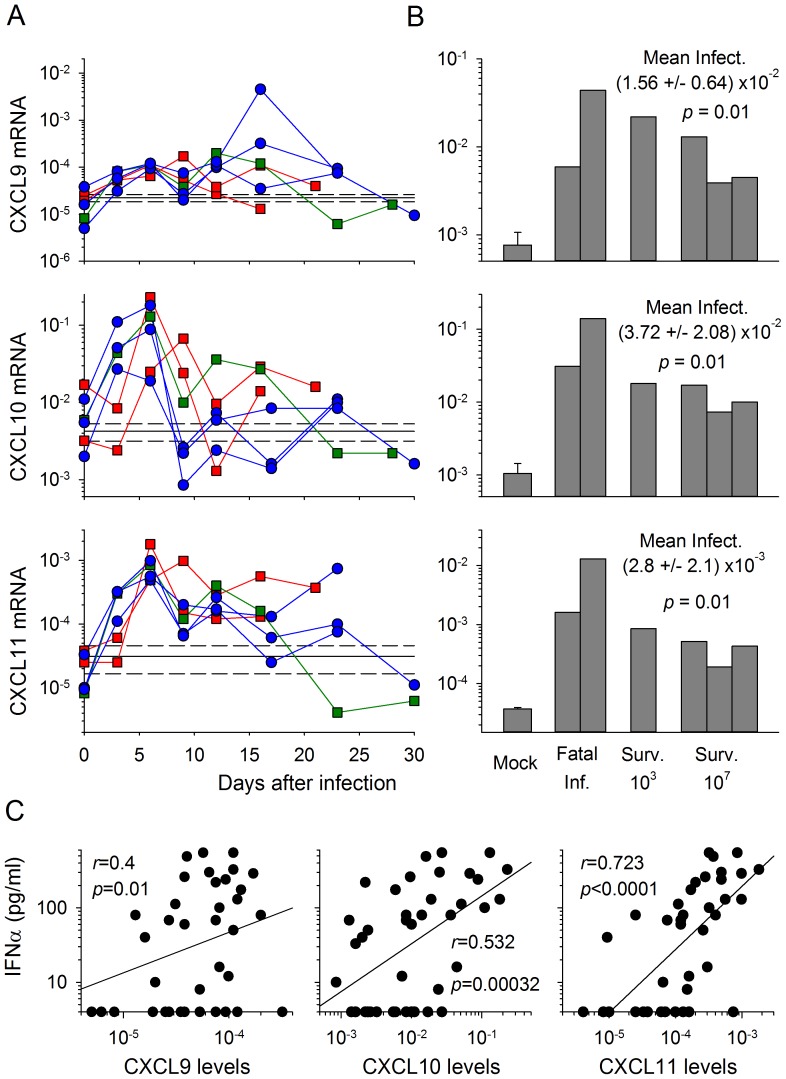
Production of CXC chemokine mRNA in PBMC and lymph nodes from LASV-infected cynomolgus monkeys. (A) The synthesis of CXC chemokine mRNA was assessed by RT-qPCR, with normalization against β-actin mRNA in PBMC obtained from fatally infected monkeys (red squares), the low dose-infected survivor (green squares) and the high dose-infected survivors (blue circles), at various times after infection. The mRNA levels in the two mock-infected animals are represented as the mean (solid line) ± SE (hashed line) of the values obtained throughout follow-up (*n = *14). (B) The production of these mRNAs was also quantified in lymph nodes obtained 9 days after infection. The levels of mRNA are represented as the mean ± SE of 4 samples for mock-infected animals (two animals, lymph nodes obtained 9 days after infection and at necropsy) and as individual values for the two fatally infected monkeys (fatal inf.), the low dose (10^3^ FFU)-infected survivor and the three high dose (10^7^ FFU)-infected survivors. The mean value ± SE for all infected animals is shown, together with the significant difference between mock- and LASV-infected animals. C) Correlation between CXCL9, CXCL10 and CXCL11 mRNA levels in PBMC and type I IFN levels in plasma during the course of LASV infection in cynomolgus monkeys, represented by a linear regression with a correlation coefficient *r* and a probability of correlation *p*.

We compared the results obtained for both PBMC and lymph nodes with those obtained for these samples from the two mock-infected monkeys. Levels of CCL2, CCL3, CCL4, CCL5 and CCL7 mRNA synthesis in the infected monkeys were no higher than those in the mock-infected monkeys (data not shown). By contrast, increases in the amounts of mRNA encoding CXCL9, CXCL10 and CXCL11 were detected in PBMC during the course of the disease, and in the lymph nodes of all infected monkeys (figure 6A and B and [17]). CXCL10 and CXCL11 mRNA synthesis in PBMC was most marked six days after infection, in all infected monkeys (figure 6A and [17]). However, three days after infection, these mRNAs were considerably more abundant in survivors than in the monkeys that subsequently died. These outcome-associated differences were no longer evident six days after infection and were not detected at any other point in the course of the disease.

Finally, the increase in mRNA levels for CXCL10 and CXCL11 and, to a lesser extent, CXCL9, in PBMC was strongly correlated with the levels of IFNα release into the plasma of the animals (as reported in [17]) during the course of LASV infection (Figure 6C).

## Discussion

Little is known about the pathogenesis of LF and the immune responses that allow a substantial number of patients to recover from acute hemorrhagic fever or even to control viral replication without developing symptoms. We have developed *in vitro* models for studying the response of human immune cells to infection with the closely related arenaviruses LASV and MOPV, for the identification of host parameters associated with virulence (LASV) or an absence of pathogenicity (MOPV) [10], [24], [25], [26], [43]. We have also recently developed reverse genetics techniques for characterizing the role of viral factors in the pathogenicity of LASV [31]. Here, we compare the abilities of LASV and MOPV to induce the production of CC and CXC chemokines in human APC, cultured alone or in the presence of T cells. Our findings provide confirmation *in vivo*, in NHP, that LF is associated with CXC chemokine release.

We found that MOPV induced a massive release of CC and, more particularly, of CXC chemokines in MP. By contrast, significant, but modest, increases in mRNA production in response to infection were observed only for CXCL10 and CXCL11 in LASV-infected MP. This striking difference in the response of MP to infections with LASV and MOPV is consistent with our previous finding that these cells are strongly activated by MOPV, but not by LASV [24], [25]. The cells potentially targeted by these chemokines express CCR1, CCR2, CCR5 and CXCR3; they are thus mostly monocytes, MP, iDC, activated T cells, Th1 cells, B cells, and NK cells [44]. The release of substantial amounts of chemokines by MOPV-infected MP may play a crucial role in the lack of pathogenicity associated with this virus. Indeed, these mediators may act at several levels in the control of viral infection. First, most of the CC chemokines described here are involved in the induction of innate responses through the recruitment of inflammatory cells, mostly iDC and MP, to the site of infection for the establishment of a local inflammatory response [45], [46], [47]. CC chemokines, such as CCL2, 3, 4, 5 and 7, are also involved in attracting naïve and activated CD4^+^ and CD8^+^ T cells and bringing them into contact with antigen-presenting DC [34], [48], [49], [50], [51]. CXCL9, CXCL10 and CXCL11 are also crucial for the induction of immune responses. Indeed, these CXC chemokines are recognized by CXCR3, a receptor specifically expressed by activated T lymphocytes and, to a lesser extent, NK cells, but not resting T cells [52], [53]. CXCL9, 10 and 11 are therefore major attractors of activated T cells, mostly Th1 and CTL, and they mediate the recruitment of effector T cells and NK cells in secondary lymphoid organs and inflamed tissues [54], [55], [56], [57]. These chemokines have also been reported to stimulate T-lymphocyte proliferation and effector cytokine production [58]. Overall, these results suggest that the production of CXC chemokines may be beneficial, helping to combat viral infection [59]. However, an involvement of these chemokines in immunopathological events has also been reported in some viral infections [38], [60], and further investigations are required to clarify the role of CXC chemokines during LF, as we found that LASV also induced the release of significant quantities of CXCL10 and 11 *in vitro* in MP and *in vivo* in NHP. The production of these mediators in lymph nodes seemed to be more elevated 9 days after infection in fatally-infected animals than in survivors. Although this difference was noticeable in only one fatality, it would be interesting to investigate whether there are higher amounts of chemokines released in secondary lymphoid organs during severe LF in comparison with non fatal infection and whether these mediators play a deleterious role in this case.

The role of chemokines in LF in humans and relevant NHP models remains unclear. Serum concentrations of IL-8 (CXCL8) and CXCL10 have been shown to be higher in patients surviving acute LF than in those who die, whereas CCL5 concentrations are high in both groups [39], suggesting a possible beneficial effect of CXC chemokines on outcome. Fatal LASV infection in cynomolgus monkeys has been associated with high plasma concentrations of CCL2 and eotaxin in the absence of CCL5, CXCL9 and CXCL10 release [11]. A transcriptomic analysis on PBMC from cynomolgus monkeys fatally infected with LASV by aerosol recently reported the upregulation of CCL23, CCRL2, IL-8 and CXCL12 mRNA synthesis [61]. We did not detect the synthesis of CC chemokines in our cynomolgus monkeys infected with LASV, which is not consistent with the results obtained with MOPV or rLASV NP389A/D392A. This discrepancy is unclear but may be linked to the lack of MP in PBMC and to their limited proportion among splenocytes, as these cells were the main source of CC chemokines *in vitro*. In any case, the putative protective role of CC chemokines in LF should be interpreted according to this apparent lack of *in vivo* production. In contrast, we did observe an increase in mRNA levels for CXCL9, 10 and 11, in both PBMC and lymph nodes (this report and [17]). No significant difference in mRNA levels during acute disease was detected between lymph nodes (9 days after infection) and PBMC (from 6 days after infection). However, very shortly after infection (3 days after infection), during the incubation period, CXCL10 and CXCL11 mRNA levels were found to be higher in the PBMC of the monkeys that subsequently survived the acute infection than in the PBMC of monkeys that died. These results have to be confirmed, as the number of animals included here was very low and because the chemokine levels have not been evaluated in lymph nodes at this early time. Nevertheless, these results are consistent with the higher levels of IL-8 (CXCL8) and CXCL10 detected in serum samples from patients surviving acute LF than in those from patients who die [39], suggesting that CXC chemokines may be beneficial for outcome, particularly during early stages of the disease. However, the evaluation of the overall production of chemokines in patients would be required to determine whether there is a correlation between the severity of LF and the production of chemokines. If it be so, it would be important to understand why different chemokine responses are induced by the same virus in patients. The mechanisms that lead to the diversity of clinical presentation of LF in humans ranging from subclinical infection to catastrophic illness and death, are not clarified. These different outcomes could be due to the type of cells early targeted by the virus, the route of infection, the inoculum dose, preexisting immunity against LASV or heterologous immunity, a different immunological status at the time of infection or a genetic background (HLA). LF was characterized by a massive, generalized infiltration of mononuclear cells, mostly macrophages, into the tissues and organs of cynomolgus monkeys [17]. These cells could potentially be the source of CXC chemokines, attracting activated T cells and NK cells to sites of inflammation. It is possible that the rapidity with which this innate response is established determines whether the disease is fatal or has a favorable outcome. However, the mechanisms leading to a rapid response in some animals and a slower response in others remain unclear, and further investigations of this aspect are required.

The production of CCL4, CCL7, CXCL10 and CXCL11 in LASV- and MOPV-infected DC cultured in the presence of autologous T cells was strongly correlated with type I IFN synthesis, and neutralization of the type I IFN receptor completely abolished the release of these chemokines. Chemokine production was inhibited during the first coculture in the presence of type I IFN receptor-neutralizing antibodies, but also after the second stimulation of T cells with MOPV-pulsed DC. The lack of type I IFN and/or chemokines during the first round of T-cell stimulation with MOPV-infected DC therefore had major consequences for the priming of T cells, altering their ability to activate iDC during the second round of stimulation. These results suggest that these mediators play a crucial role in the induction of adaptive T-cell responses to MOPV and provide additional evidence in favor of a beneficial role of chemokines during arenavirus infection. The ability of type I IFN to stimulate the production of CXC chemokines, such as CXCL10 and CXCL11 in particular, is well documented [62]. This close link between type I IFN and CXC chemokine production was also observed *in vivo*, in our primate model. In particular, the early release of IFNα observed specifically in the plasma of the monkeys that survived and not in those that died [17], is consistent with the increase in CXCL10 and CXCL11 mRNA levels in PBMC. The different production of type I IFN observed between surviving and fatally-infected monkeys despite the presence in animals of a virus presenting similar ability to inhibit this response is unclear. One hypothesis could be that the early type I IFN release observed in surviving primates was due to plasmacytoid DC (pDC), as pDC activation following viral infection is mediated by different TLR than myeloid DC or MP. Indeed, the ability of mouse pDC to bind LASV GP and to release type I IFN after infection with LCMV has been recently reported [63], suggesting that these cells could play a crucial role in the induction of immune responses during LF. Further investigations are required to confirm this hypothesis *in vivo* and, if it occurs, to explain the activation of these cells in some animals only. In addition, it would be important to evaluate the ability of pDC to produce chemokines after arenavirus infection to better understand the role of these cells during infection. Nevertheless, these results suggest that the different responses of APC to MOPV and LASV make a major contribution to the difference in pathogenicity between these viruses and to the crucial role of an early activation of innate immunity to the induction of a successful adaptive response and survival in LF. Although the rLASV NP-D389A/G392A is severely attenuated in APC [31], LASV and MOPV replicate at similar levels in APC [10], [25], suggesting that the different chemokine responses are not related to different amounts of viral particles released. Further evidence of the correlation between type I IFN and chemokine responses was provided by the analysis of APC responses to infection with our rLASV NP-D389A/G392A. This LASV bears mutations abolishing the function of the exonuclease domain of the NP; it therefore strongly induces type I IFN production [31]. Consequently, the levels of production of CC and CXC chemokines by MP infected with this virus reached those observed with MOPV. More interestingly, whereas only modest levels of CXC chemokines and no significant synthesis of CC chemokines were induced in MOPV-infected DC, large amounts of mRNAs encoding all these chemokines were observed in response to the rLASV. Despite the presence of the exonuclease site, which is conserved among arenavirus [64], in MOPV NP, this virus is however able to induce substantial type I IFN responses in APC. Several hypotheses can explain this observation. Other inhibitory properties have been linked to arenavirus NP, such as the ability to sequester IKKε in an inactive form or to inhibit NFκB activation [32], [33]. It is therefore possible that the efficiency of the exonucleases to digest RNA or of these other inhibitory properties of the NP is different between both viruses. In addition, the role of other viral proteins such as the Z protein in immunosuppression cannot be excluded. These results confirm the central role of NP in the pathogenicity and lack of immunogenicity of LASV, and demonstrate that the ability of LASV NP to inhibit type I IFN induction has profound consequences for the innate, and probably also adaptive, immune responses directed against LASV.

In summary, we show here, in both *in vitro* and *in vivo* models, that CC and CXC chemokines, including CXCL10 and CXCL11 in particular, are probably key mediators during LF. Our results suggest that the production of these molecules is associated with the lack of pathogenicity of MOPV and with the immunogenicity of LASV NP-D389A/G392A, and that their early release during the incubation period is associated with the control of LASV infection in NHP. Finally, we show that APC must produce type I IFN if they are to release chemokines, and this requirement for type I IFN seems to hold for cynomolgus monkeys. Further investigations in NHP will nevertheless be required, to confirm the protective role of these mediators and to elucidate their mode of action.
